# Effect of submucosal application of tramadol on postoperative pain after third molar surgery

**DOI:** 10.1186/s13005-015-0090-9

**Published:** 2015-10-14

**Authors:** Onur Gönül, Tülin Satılmış, Ferit Bayram, Gökhan Göçmen, Aysegül Sipahi, Kamil Göker

**Affiliations:** Oral and Maxillofacial Surgery Department, Faculty of Dentistry, Marmara University, Istanbul, Turkey

**Keywords:** Tramadol, Third molar surgery, Postoperative analgesia

## Abstract

The aim of this study was to evaluate the effectiveness of submucosal application of tramadol, for acute postoperative facial pain, following the extraction of impacted third molar teeth. This prospective, double-blind, randomised placebo-controlled study included 60 ASA I-II patients undergoing impacted third molar surgery under local anaesthesia. Following the surgical procedure, patients were randomly divided into two groups; group T (1 mg/kg tramadol) and group S (2-mL saline). Treatments were applied submucosally after surgery. Pain after extraction was evaluated using a visual analogue scale (VAS) 0.5, 1, 2, 4, 6, 12, 24, and 48 h postoperatively. The time at which the first analgesic drug was taken, the total analgesic dose used, and adverse tissue reactions were also evaluated. In group T, postoperative VAS scores were significantly lower compared to that in group S (*p* < 0.05). This study demonstrated that post-operative submucosal application of tramadol is an effective method for reducing acute post-operative facial pain after impacted third molar surgery.

## Introduction

Tramadol hydrochloride is a centrally acting synthetic opioid analgesic. Although classified as a weak opioid in terms of its analgesic properties, tramadol exerts a double action, functioning as both an opioid and a non-opioid. It also reduces the transmission of pain impulses by inhibiting serotonin and norepinephrine re-uptake [1], thereby inducing a combined analgesic/adjuvant effect. The incidence of opioid abuse is very high; the main side-effects are nausea, vomiting, drowsiness and dizziness. Opioids also exert a depressive effect on the cardiovascular and respiratory systems [2–4]. Although it is an opioid, tramadol produces few side-effects; therefore it can be used for both acute and chronic pain.

Surgical extraction of wisdom teeth is among the most-frequently performed dental surgical procedures, and is associated with medium-severe pain. Between 3–5 h after surgery, the efficacy of local anaesthesia declines and pain reaches its maximum level [1]. Therefore, for many years researchers have aimed to identify a more-effective analgesic for application after surgical tooth extraction. The purpose of this study was to evaluate the effectiveness of submucosal application of tramadol, for acute postoperative facial pain, following the extraction of impacted third molar teeth.

## Materials & methods

This comparative, prospective, randomised study enrolled 60 patients from the Department of Oral and Maxillofacial Surgery, Dentistry Faculty, Marmara University, Istanbul, Turkey. Ethics Committee approval was obtained from the appropriate institution (Yeditepe University Local Ethics Committee. Istanbul-Turkey. Approval no. 155/6122011). Informed consent was obtained from all participants.

To standardise the study, both groups comprised elective patients who had undergone unilateral mandibular third molar extraction surgery. All patients were > 18 years of age, weighed < 100 kg and were classified as ASA I-II using the guidelines of the American Society of Anesthesiology [5]. The study used a randomized, double-blind, placebo-controlled design. The exclusion criteria were use of sedatives, tranquilisers, or analgesic drugs 24 h before treatment, a history of sensitivity to tramadol, and the use of more than three ampoules of local anaesthesia during the procedure. All of the teeth included to study were mesially angulated and completely impacted third molars. A simple randomization technique was used to achieve randomization by using a random number generator.

During surgery, all patients were in the semi-supine position. Electrocardiography, non-invasive blood pressure and peripheral oxygen saturation monitors were firstly attached. Then, an inferior dental nerve block was obtained using 4 % articaine HCl with 1:100,000 epinephrine HCl (Ultracaine D-S Forte; Aventis, Bridgewater, NJ, USA). The efficacy of the local anaesthetic was assessed by verbal questioning and gentle probing of the buccal and lingual surfaces of the impacted mandibular third molar which was removed using standard surgical techniques.

After the extraction of the tooth, patients were randomly divided into two groups: group T (1 mg/kg tramadol diluted with saline to 2 mL) and group K (2-mL saline). The 2 mL volume of solutions were prepared by an anaesthetic nurse and placed in sterile disposable syringes. Both the surgeon and the patients were blinded to the specific solution used. After extraction of the tooth, the surgeon applied the solution to the extraction socket and the bone surface by means of small drops. The surgeon and the patients were both blinded to the specific solution used. The time at which the local anaesthesia was applied was defined as time 0; the time at which the extraction procedure started, and the total extraction time, were also recorded.

The postoperative mean blood pressure, heart rate, peripheral oxygen saturation and Ramsay scores of all patients were recorded at 10-min intervals. Patients were questioned about side-effects (burning, nausea, vomiting, weakness and hallucinations) after the procedure.

Pain after extraction was evaluated using a visual analogue scale (VAS); patients were asked to score overall pain at 0.5, 1, 2, 4, 6, 12, 24 and 48 h using the VAS (0, no pain; 5, pain requiring analgesia; 10, excessive pain). Patients were asked to record the time and amount of analgesic taken after surgical extraction; total analgesic consumption during the first 48 h was also recorded. Data charts were collected from patients at the end of the follow up period.

The data were analysed using the SPSS for Windows software package (ver. 16.0; SPSS Inc., Chicago, IL, USA). Descriptive statistics (mean, standard deviation, frequency) were obtained and the chi-squared test was used to compare groups.

## Results

A total of 60 patients was included in this study (30 in each group). The distribution of patients among groups is shown in Table 1. There were no significant group differences in grouping variables, such as the difficulty of the procedure or duration of extraction. No complications were associated with the extraction procedure. The VAS scores of the control group (group S) 1, 2, 4, 6 and 12 h postoperatively, were significantly higher compared to the tramadol group (group T). There were no significant group differences in VAS scores 24 and 48 h postoperatively (*p* > 0.05). The first analgesic was taken significantly later in the tramadol group compared to the control group (*p* = 0.0001). Total analgesic intake in the control group was significantly higher (*p* = 0.0001; Table 1). Pain peak time for group S was 1 h after operation and for group T was 4 h after operation (Table 1).

**Table 1:** Patient characteristics, surgery time, total surgery time, visual analogue scale (VAS) scores, analgesic intake

	Group S			Group T				
	Mean	±	SD	Mean	±	SD	Test Value	*p*
Age (years)	23.93	±	2.828	24.80	±	2.524	−1.252	0.215^a^
Height (cm)	168.13	±	8.033	168.23	±	8.705	−0.074	0.941^b^
Weight (kg)	65.00	±	10.286	64.93	±	11.453	−0.603	0.547^b^
ASA Grade	1.00	±	0.000	1.00	±	0.000		
Total Surgery Time (min)	48.00	±	4.185	46.90	±	4.528	−1.141	0.254^b^
Surgery Time (min)	26.27	±	3.591	25.37	±	3.306	1.01	0.317^a^
VAS 0.5 h	0.00	±	0.000	0.00	±	0.000		
VAS 1 h	6.37	±	0.850	2.23	±	0.898	−6.755	0.0001^b^
VAS 2 h	3.63	±	0.615	1.87	±	0.819	−6.188	0.0001^b^
VAS 4 h	3.07	±	0.691	4.30	±	1.264	−4.714	0.0001^b^
VAS 6 h	3.33	±	1.446	1.77	±	1.382	−3.849	0.0001^b^
VAS 12 h	0.63	±	0.765	1.17	±	0.791	−2.668	0.008^b^
VAS 24 h	0.23	±	0.430	0.10	±	0.305	−1.374	0.169^b^
VAS 48 h	0.07	±	0.254	0.00	±	0.000	−1.426	0.154^b^
Initial Analgesic Intake	1.87	±	0.681	4.97	±	0.809	−6.779	0.0001^b^
Total Analgesic Intake	3.60	±	0.814	1.67	±	0.547	−6.47	0.0001^b^

^a^Student’s *t*-test (test value “t”)

^b^Mann-Whitney *U* test (test value “Z”)

There were no significant group differences in side-effects (nausea, vomiting, burning, and dizziness; Table 2). Mean blood pressure, heart rate and peripheral oxygen saturation are displayed in Table 3. Although there were significant differences in mean blood pressure and heart rate between 0 and 30 min, these differences were not clinically significant.

**Table 2:** Patient characteristics and side-effects

						Test Value	df	*p* ^a^
		Group S		Group T				
		*n*	%	*n*	%			
Gender	Female	17	56.70	17	56.70	0.000	1	1.000
	Male	13	43.30	13	43.30			
Accompanying Disease	Absent	30	100.00	30	100.00			
	Present	0	0.00	0	0.00			
Nausea	Absent	28	93.30	25	83.30	1.456	1	0.228
	Present	2	6.70	5	16.70			
Vomiting	Absent	28	93.30	30	100.00	2.069	1	0.150
	Present	2	6.70	0	0.00			
Burning	Absent	27	90.00	30	100.00	3.158	1	0.076
	Present	3	10.00	0	0.00			
Dizziness	Absent	30	100.00	30	100.00			
	Present	0	0.00	0	0.00			

^a^Pearson’s chi-squared test

**Table 3:** Vital Signs of Groups

	Group S			Group T				
	Mean	±	SD	Mean	±	SD	Test Value	*p*
MBP 0. min	60,10	±	1,539	61,13	±	1,717	−2,455	0,017^a^
MBP 10. min	60,77	±	1,478	60,73	±	1,337	−0,008	0,994^b^
MBP 20. min	60,37	±	1,450	60,50	±	1,009	−0,183	0,855^b^
MBP 30. min	60,60	±	1,276	61,00	±	0,910	−1,536	0,125^b^
MBP 40. min	60,60	±	1,404	61,03	±	1,299	−1,24	0,220^a^
MBP 50. min	60,47	±	1,306	60,77	±	1,382	−0,864	0,391^a^
MBP 60. min	60,57	±	1,455	60,73	±	1,413	−0,45	0,654^a^
HR 0. min	73,37	±	2,988	73,00	±	2,994	0,475	0,637^a^
HR 10. min	73,67	±	3,427	73,27	±	3,129	0,472	0,639^a^
HR 20. min	73,97	±	3,306	73,83	±	3,485	0,152	0,880^a^
HR 30. min	74,27	±	3,183	72,70	±	2,215	2,213	0,031^a^
HR 40. min	74,10	±	3,377	73,07	±	3,084	1,238	0,221^a^
HR 50. min	74,27	±	3,084	72,90	±	2,998	1,74	0,087^a^
HR 60. min	74,10	±	3,033	74,17	±	2,829	−0,088	0,930^a^
SpO2 0. min	98,73	±	0,450	98,80	±	0,407	−0,605	0,545^b^
SpO2 10. min	98,87	±	0,346	98,77	±	0,430	−0,993	0,321^b^
SpO2 20. min	98,87	±	0,346	98,70	±	0,466	−1,554	0,120^b^
SpO2 30. min	98,80	±	0,407	98,80	±	0,407	0	1,000^b^
SpO2 40. min	98,77	±	0,430	98,63	±	0,490	−1,117	0,264^b^
SpO2 50. min	98,83	±	0,379	98,73	±	0,450	−0,932	0,351^b^
SpO2 60. min	98,80	±	0,407	98,73	±	0,450	−0,605	0,545^b^

^a^Student’s *t*-test (test value “t”)

^b^Mann-Whitney *U* test (test value “Z”)

*MBP* Mean Blood Pressure, *HR* Heart Rate, *SpO2* Pheripheral Oxygen Saturation

## Discussion

Following impacted third molar surgery, medium-severe pain occurs during the early postoperative stage. To improve patient satisfaction after dental surgical procedures, postoperative pain should be reduced. Several studies have assessed the effectiveness of tramadol application for analgesia after surgery, but few have evaluated submucosal application. In one study, submucosal tramadol was administered after paediatric tonsillectomy surgery, which reduced the need for post-surgical analgesia [6]. In another study, a combination of tramadol and acetaminophen tablets was used and conferred highly effective analgesia [7]. Collins et al. performed a study assessing the effect of tramadol for the relief of pain after dentoalveolar operations. The operations included bone removal and suturing. At the end of the study, it has been shown that tramadol was successful in complete pain relief which was continued for the following 2 days [2]. In a study, they investigated the analgesic effects of tramadol. Tramadol was given systemically, applied into the surgical site after the extraction of impacted third molar under local anesthesia. It has been found that tramadol extends the duration of anesthesia in both local and systemical administration, and shown that it improves the quality of postoperative analgesia [8]. In a comparative study, combinations of 10-mg oral ketorolac and 50-mg submucosal tramadol, and 10-mg oral ketorolac and saline, were used. The treatments were administered 30 min before the impacted third molar surgery; combination tramadol treatment was more effective in reducing post-operative pain and the total amount of analgesic required [9]. In a comparative study by Ong et al. (2005), the effectiveness of intravenous (i.v.) and oral tramadol, after impacted third molar surgery, was compared in patients under sedation (performed using midazolam). VAS scores were significantly lower, analgesic intake time was later, and total analgesic intake 48 h post-operatively was reduced, in the i.v. group. Furthermore, single-dose oral tramadol was insufficient for analgesia after impacted tooth surgery [4]. Another comparative, prospective, randomised double-blind study, by Pozos et al. (2007), compared preoperative and postoperative 100-mg intramuscular tramadol. Preoperative tramadol was more effective in reducing postoperative pain [10]. In an another study, Isıordia Espinoza MA and et al. compared the pre-emptive analgesic effectiveness of 15 mg of meloxicam and 50 mg of tramadol after mandibular third molar surgery at the end of the study they revealed that the patients receiving 15 mg of preoperative meloxicam had less pain intensity and total analgesic consumption than those receiving 50 mg of preoperative tramadol [11]. Another double-blind, randomized, placebo-controlled, crossover clinical trial by Isıordia Espinoza MA and et al. was performed to evaluate the effect of submucous tramadol as adjuvant of mepivacaine with epinephrine in inferior alveolar nerve block and stated that submucous tramadol increased the anesthetic efficacy of mepivacaine with epinephrine of soft tissue in inferior alveolar nerve block [12]. Ceccheti MM and et al., conducted a study to evaluate the analgesic and adjuvant anesthetic effects of submucosal tramadol after third molar extraction. They found that submucosal tramadol injection after oral surgery improved postoperative analgesia, but did not extend anesthetic action duration [13]. In the present study, VAS scores at 1, 2, 4, 6 and 12 h were significantly lower, first analgesic intake was significantly later, and total analgesic intake was significantly lower, in the tramadol group compared to controls.

## Conclusion

Submucosal tramadol represents an effective, safe and reliable method of reducing postoperative acute facial pain after impacted third molar surgery. However, further studies are required to validate the efficacy of submucosal tramadol after dental or surgical procedures.
